# 3-[(*E*)-(4-Chloro­benzyl­idene)amino]-1-phenyl­thio­urea

**DOI:** 10.1107/S1600536811008920

**Published:** 2011-03-12

**Authors:** Nur Nadia Dzulkifli, Yang Farina, Bohari M. Yamin, Ibrahim Baba, Edward R. T. Tiekink

**Affiliations:** a School of Chemical Sciences and Food Technology, Faculty of Science and Technology, Universiti Kebangsaan Malaysia, 43600 Bangi, Malaysia; b Department of Chemistry, University of Malaya, 50603 Kuala Lumpur, Malaysia

## Abstract

In the title compound, C_14_H_12_ClN_3_S, the dihedral angle between the terminal benzene rings is 56.6 (2)°; the benzene rings lie to the same side of the mol­ecule. The major twist in the mol­ecule occurs around the C_ar_—N bond (ar is aromatic) [C—N—C—C = 49.9 (5)°]. The configuration about the N=C bond [1.271 (4) Å] is *E*. The amine H atoms lie on opposite sides of the mol­ecule with one forming an intra­molecular N—H⋯N(imine) hydrogen bond and an *S*(5) ring. In the crystal, centrosymmetric dimers are formed *via* {⋯HNC=S}_2_ synthons.

## Related literature

For related structures, see: Cunha *et al.* (2007 ▶); Kayed *et al.* (2008 ▶).
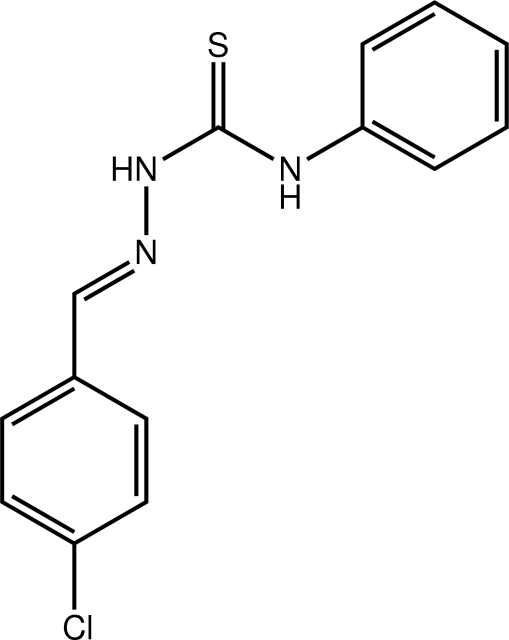


## Experimental

### 

#### Crystal data

C_14_H_12_ClN_3_S
*M* *_r_* = 289.78
Monoclinic, 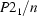

*a* = 15.082 (5) Å
*b* = 6.560 (2) Å
*c* = 15.205 (5) Å
β = 110.272 (8)°
*V* = 1411.2 (8) Å^3^
*Z* = 4
Mo *K*α radiation
μ = 0.41 mm^−1^
*T* = 293 K
0.39 × 0.21 × 0.02 mm

#### Data collection

Oxford Diffraction Xcaliber Eos Gemini diffractometer
Absorption correction: multi-scan (*CrysAlis PRO*; Oxford Diffraction, 2010 ▶) *T*_min_ = 0.857, *T*_max_ = 0.992
8647 measured reflections
2905 independent reflections
1572 reflections with *I* > 2σ(*I*)
*R*_int_ = 0.080

#### Refinement

*R*[*F* ^2^ > 2σ(*F* ^2^)] = 0.068
*wR*(*F* ^2^) = 0.137
*S* = 1.00
2905 reflections
178 parameters
2 restraints
H atoms treated by a mixture of independent and constrained refinement
Δρ_max_ = 0.32 e Å^−3^
Δρ_min_ = −0.21 e Å^−3^

### 

Data collection: *CrysAlis PRO* (Oxford Diffraction, 2010 ▶); cell refinement: *CrysAlis PRO*; data reduction: *CrysAlis PRO*; program(s) used to solve structure: *SHELXS97* (Sheldrick, 2008 ▶); program(s) used to refine structure: *SHELXL97* (Sheldrick, 2008 ▶); molecular graphics: *ORTEP-3* (Farrugia, 1997 ▶) and *DIAMOND* (Brandenburg, 2006 ▶); software used to prepare material for publication: *publCIF* (Westrip, 2010 ▶).

## Supplementary Material

Crystal structure: contains datablocks global, I. DOI: 10.1107/S1600536811008920/hb5814sup1.cif

Structure factors: contains datablocks I. DOI: 10.1107/S1600536811008920/hb5814Isup2.hkl

Additional supplementary materials:  crystallographic information; 3D view; checkCIF report

## Figures and Tables

**Table 1:** Hydrogen-bond geometry (Å, °)

*D*—H⋯*A*	*D*—H	H⋯*A*	*D*⋯*A*	*D*—H⋯*A*
N1—H1*n*⋯N3	0.85 (2)	2.16 (4)	2.601 (4)	112 (3)
N2—H2*n*⋯S1^i^	0.86 (3)	2.57 (3)	3.401 (3)	164 (2)

Symmetry code: (i) 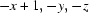.

**Fig. 1.**
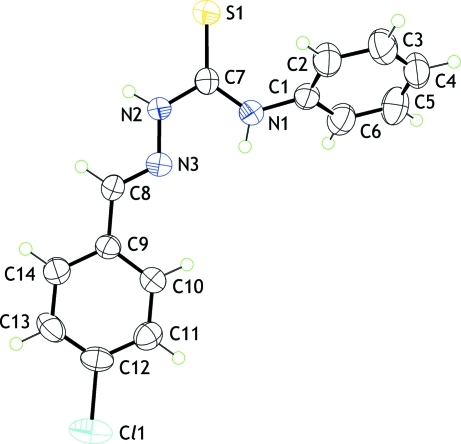
The molecular structure of of (I) showing displacement ellipsoids at the 50% probability level.

**Fig. 2.**
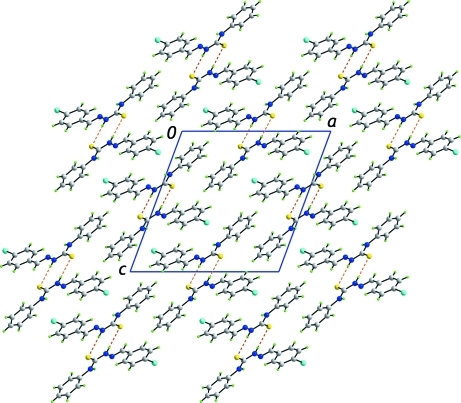
A view in projection down the b axis of the unit-cell contents for (I) showing the packing: the N–H···S hydrogen bonds are shown as orange dashed lines.

## supplementary crystallographic information

### Comment

The title compound, (I), was investigated in continuation of structural studies of substituted thiosemicarbazone molecules (Kayed *et al.*, 2008). The configuration about the N3═C8 bond [1.271 (4) Å] in (I), Fig. 1, is *E*. The C1-benzene ring is twisted out of the plane of the thiourea moiety as seen in the value of the C7—N1—C1—C2 torsion angle of 49.9 (5) °; the dihedral angle between the C7,N1,N2,S1 [r.m.s. = 0.0155 Å] and C1–C6 planes is 51.06 (13) °. By contrast, the C9-benzene ring is co-planar with the imine moiety, with N3—C8—C9—C10 being -6.3 (5) °. The dihedral angle formed between the benzene rings is 56.6 (2) °. The amine groups lie on opposite sides of the molecule allowing for the formation of an intramolecular N1—H1*n*···N3 hydrogen bond, Table 1. The result is that the benzene rings lie to the same side of the molecule. Overall the conformation of the molecule is a twisted U-shape. The geometric parameters in and the overall conformation of (I) match very closely those found in the unsubstituted parent compound (Cunha *et al.*, 2007).

The major feature of the crystal packing is the formation of centrosymmetric dimers *via* N—H···S hydrogen bonds, Table 1 and Fig. 2.

### Experimental

The title compound was prepared by heating an ethanolic (50 ml) solution of 4-chlorobenzaldehyde (1.406 g, 10 mmol) and 4-phenylthiosemicarbazide (1.672 g, 10 mmol) under reflux for 1 h. The resulting product was isolated and recrystallized from DMSO to afford light pink plates in 72% yield (*M*.pt. 470–472 K). IR (KBr): Strong absorption bands for thiocarbonyl ν(C═S), azomethine ν(C═N) and hydrazinic nitrogen ν(N—N) appeared at 1199, 1600 and 1083 cm^-1^, respectively. Absorption bands for ν(C—Cl) and aromatic carbon ν(C═C) appeared at 833 and 1510 cm^-1^, respectively.

### Refinement

Carbon-bound H-atoms were placed in calculated positions (C—H 0.93 Å) and were included in the refinement in the riding model approximation, with *U*_iso_(H) set to 1.2 to 1.5*U*_equiv_(C). The N-bound H atoms were refined with the distance restraint 0.86±0.01 Å with *U_iso_*(H) = 1.2*U_eq_*(parent atom).

### Crystal data

**Table:**

C_14_H_12_ClN_3_S	*F*(000) = 600
*M**_r_* = 289.78	*D*_x_ = 1.364 Mg m^−^^3^
Monoclinic, *P*2_1_/*n*	Mo *K*α radiation, λ = 0.71073 Å
Hall symbol: -P 2yn	Cell parameters from 752 reflections
*a* = 15.082 (5) Å	θ = 2.4–19.2°
*b* = 6.560 (2) Å	µ = 0.41 mm^−^^1^
*c* = 15.205 (5) Å	*T* = 293 K
β = 110.272 (8)°	Plate, light-pink
*V* = 1411.2 (8) Å^3^	0.39 × 0.21 × 0.02 mm
*Z* = 4	

### Data collection

**Table:**

Oxford Diffraction Xcaliber Eos Gemini diffractometer	2905 independent reflections
Radiation source: fine-focus sealed tube	1572 reflections with *I* > 2σ(*I*)
graphite	*R*_int_ = 0.080
Detector resolution: 16.1952 pixels mm^-1^	θ_max_ = 26.5°, θ_min_ = 1.6°
ω scans	*h* = −17→18
Absorption correction: multi-scan (*CrysAlis PRO*; Oxford Diffraction, 2010)	*k* = −8→8
*T*_min_ = 0.857, *T*_max_ = 0.992	*l* = −17→19
8647 measured reflections	

### Refinement

**Table:**

Refinement on *F*^2^	Primary atom site location: structure-invariant direct methods
Least-squares matrix: full	Secondary atom site location: difference Fourier map
*R*[*F*^2^ > 2σ(*F*^2^)] = 0.068	Hydrogen site location: inferred from neighbouring sites
*wR*(*F*^2^) = 0.137	H atoms treated by a mixture of independent and constrained refinement
*S* = 1.00	*w* = 1/[σ^2^(*F*_o_^2^) + (0.0511*P*)^2^] where *P* = (*F*_o_^2^ + 2*F*_c_^2^)/3
2905 reflections	(Δ/σ)_max_ < 0.001
178 parameters	Δρ_max_ = 0.32 e Å^−^^3^
2 restraints	Δρ_min_ = −0.21 e Å^−^^3^

### Special details

**Table:**

Geometry. All s.u.'s (except the s.u. in the dihedral angle between two l.s. planes) are estimated using the full covariance matrix. The cell s.u.'s are taken into account individually in the estimation of s.u.'s in distances, angles and torsion angles; correlations between s.u.'s in cell parameters are only used when they are defined by crystal symmetry. An approximate (isotropic) treatment of cell s.u.'s is used for estimating s.u.'s involving l.s. planes.
Refinement. Refinement of *F*^2^ against ALL reflections. The weighted *R*-factor *wR* and goodness of fit *S* are based on *F*^2^, conventional *R*-factors *R* are based on *F*, with *F* set to zero for negative *F*^2^. The threshold expression of *F*^2^ > 2σ(*F*^2^) is used only for calculating *R*-factors(gt) *etc*. and is not relevant to the choice of reflections for refinement. *R*-factors based on *F*^2^ are statistically about twice as large as those based on *F*, and *R*- factors based on ALL data will be even larger.

### Fractional atomic coordinates and isotropic or equivalent isotropic displacement parameters (Å^2^)

**Table:**

	*x*	*y*	*z*	*U*_iso_*/*U*_eq_	
Cl1	0.89088 (7)	1.20521 (16)	0.14891 (8)	0.0759 (4)	
S1	0.43050 (7)	−0.03791 (15)	0.10962 (7)	0.0525 (3)	
N1	0.4748 (2)	0.3287 (5)	0.1918 (2)	0.0506 (8)	
H1n	0.503 (2)	0.441 (3)	0.192 (3)	0.061*	
N2	0.5408 (2)	0.2474 (4)	0.0834 (2)	0.0440 (8)	
H2n	0.548 (2)	0.170 (4)	0.0405 (18)	0.053*	
N3	0.58808 (19)	0.4293 (4)	0.1032 (2)	0.0420 (7)	
C1	0.4240 (2)	0.3118 (6)	0.2544 (3)	0.0466 (9)	
C2	0.4352 (3)	0.1467 (6)	0.3130 (3)	0.0602 (11)	
H2	0.4743	0.0395	0.3097	0.072*	
C3	0.3891 (3)	0.1397 (7)	0.3761 (3)	0.0720 (13)	
H3	0.3972	0.0284	0.4159	0.086*	
C4	0.3307 (3)	0.2969 (8)	0.3807 (3)	0.0710 (13)	
H4	0.2992	0.2918	0.4235	0.085*	
C5	0.3189 (3)	0.4607 (8)	0.3223 (4)	0.0758 (13)	
H5	0.2785	0.5661	0.3244	0.091*	
C6	0.3670 (3)	0.4695 (6)	0.2603 (3)	0.0632 (11)	
H6	0.3607	0.5833	0.2222	0.076*	
C7	0.4841 (2)	0.1899 (5)	0.1310 (2)	0.0409 (8)	
C8	0.6458 (2)	0.4670 (5)	0.0611 (3)	0.0433 (9)	
H8	0.6524	0.3738	0.0177	0.052*	
C9	0.7018 (2)	0.6534 (5)	0.0794 (2)	0.0396 (8)	
C10	0.6886 (2)	0.8062 (5)	0.1361 (2)	0.0440 (9)	
H10	0.6402	0.7943	0.1605	0.053*	
C11	0.7458 (2)	0.9755 (6)	0.1570 (2)	0.0497 (10)	
H11	0.7366	1.0773	0.1955	0.060*	
C12	0.8168 (2)	0.9922 (5)	0.1201 (3)	0.0476 (9)	
C13	0.8311 (3)	0.8459 (6)	0.0628 (3)	0.0549 (11)	
H13	0.8791	0.8599	0.0380	0.066*	
C14	0.7732 (2)	0.6764 (6)	0.0421 (3)	0.0529 (10)	
H14	0.7821	0.5762	0.0027	0.063*	

### Atomic displacement parameters (Å^2^)

**Table:**

	*U*^11^	*U*^22^	*U*^33^	*U*^12^	*U*^13^	*U*^23^
Cl1	0.0720 (7)	0.0538 (7)	0.0900 (9)	−0.0255 (5)	0.0129 (6)	0.0056 (6)
S1	0.0613 (6)	0.0469 (6)	0.0554 (6)	−0.0150 (5)	0.0281 (5)	−0.0079 (5)
N1	0.056 (2)	0.045 (2)	0.062 (2)	−0.0107 (15)	0.0344 (17)	−0.0113 (18)
N2	0.0534 (18)	0.0379 (19)	0.048 (2)	−0.0114 (14)	0.0273 (16)	−0.0095 (14)
N3	0.0434 (16)	0.0386 (18)	0.0456 (18)	−0.0051 (14)	0.0173 (15)	−0.0023 (14)
C1	0.042 (2)	0.050 (2)	0.050 (2)	−0.0068 (18)	0.0194 (18)	−0.011 (2)
C2	0.068 (3)	0.061 (3)	0.060 (3)	0.009 (2)	0.032 (2)	0.004 (2)
C3	0.095 (3)	0.072 (3)	0.061 (3)	0.001 (3)	0.043 (3)	0.009 (2)
C4	0.073 (3)	0.092 (4)	0.061 (3)	−0.012 (3)	0.040 (2)	−0.012 (3)
C5	0.077 (3)	0.082 (4)	0.082 (3)	0.017 (3)	0.045 (3)	−0.011 (3)
C6	0.073 (3)	0.058 (3)	0.065 (3)	0.006 (2)	0.032 (2)	−0.005 (2)
C7	0.040 (2)	0.043 (2)	0.041 (2)	0.0024 (16)	0.0163 (17)	0.0000 (18)
C8	0.050 (2)	0.040 (2)	0.047 (2)	−0.0006 (18)	0.0249 (18)	−0.0053 (18)
C9	0.044 (2)	0.035 (2)	0.041 (2)	−0.0015 (16)	0.0162 (17)	0.0026 (17)
C10	0.044 (2)	0.044 (2)	0.046 (2)	−0.0034 (17)	0.0181 (18)	0.0005 (19)
C11	0.058 (2)	0.047 (2)	0.042 (2)	0.0007 (19)	0.0145 (19)	−0.0001 (19)
C12	0.047 (2)	0.037 (2)	0.052 (2)	−0.0047 (17)	0.0082 (19)	0.0098 (18)
C13	0.053 (2)	0.049 (3)	0.073 (3)	−0.0016 (19)	0.035 (2)	0.014 (2)
C14	0.062 (3)	0.041 (2)	0.065 (3)	−0.0015 (18)	0.034 (2)	−0.002 (2)

### Geometric parameters (Å, °)

**Table:**

Cl1—C12	1.748 (4)	C4—H4	0.9300
S1—C7	1.676 (4)	C5—C6	1.374 (5)
N1—C7	1.339 (4)	C5—H5	0.9300
N1—C1	1.419 (4)	C6—H6	0.9300
N1—H1n	0.853 (10)	C8—C9	1.457 (4)
N2—C7	1.352 (4)	C8—H8	0.9300
N2—N3	1.369 (4)	C9—C10	1.381 (4)
N2—H2n	0.863 (10)	C9—C14	1.387 (4)
N3—C8	1.271 (4)	C10—C11	1.374 (4)
C1—C6	1.368 (5)	C10—H10	0.9300
C1—C2	1.375 (5)	C11—C12	1.375 (5)
C2—C3	1.366 (5)	C11—H11	0.9300
C2—H2	0.9300	C12—C13	1.363 (5)
C3—C4	1.374 (6)	C13—C14	1.381 (5)
C3—H3	0.9300	C13—H13	0.9300
C4—C5	1.366 (6)	C14—H14	0.9300
			
C7—N1—C1	128.3 (3)	N1—C7—N2	114.6 (3)
C7—N1—H1n	114 (3)	N1—C7—S1	125.6 (3)
C1—N1—H1n	117 (3)	N2—C7—S1	119.8 (3)
C7—N2—N3	120.2 (3)	N3—C8—C9	121.4 (3)
C7—N2—H2n	121 (2)	N3—C8—H8	119.3
N3—N2—H2n	119 (2)	C9—C8—H8	119.3
C8—N3—N2	117.1 (3)	C10—C9—C14	118.4 (3)
C6—C1—C2	119.5 (4)	C10—C9—C8	121.9 (3)
C6—C1—N1	118.9 (4)	C14—C9—C8	119.6 (3)
C2—C1—N1	121.5 (3)	C11—C10—C9	121.1 (3)
C3—C2—C1	120.2 (4)	C11—C10—H10	119.5
C3—C2—H2	119.9	C9—C10—H10	119.5
C1—C2—H2	119.9	C10—C11—C12	119.0 (4)
C2—C3—C4	120.1 (4)	C10—C11—H11	120.5
C2—C3—H3	119.9	C12—C11—H11	120.5
C4—C3—H3	119.9	C13—C12—C11	121.5 (3)
C5—C4—C3	119.9 (4)	C13—C12—Cl1	119.6 (3)
C5—C4—H4	120.0	C11—C12—Cl1	118.8 (3)
C3—C4—H4	120.0	C12—C13—C14	119.0 (3)
C4—C5—C6	119.9 (4)	C12—C13—H13	120.5
C4—C5—H5	120.1	C14—C13—H13	120.5
C6—C5—H5	120.1	C13—C14—C9	120.9 (4)
C1—C6—C5	120.4 (4)	C13—C14—H14	119.5
C1—C6—H6	119.8	C9—C14—H14	119.5
C5—C6—H6	119.8		
			
C7—N2—N3—C8	174.7 (3)	N3—N2—C7—S1	−177.2 (2)
C7—N1—C1—C6	−134.1 (4)	N2—N3—C8—C9	−178.1 (3)
C7—N1—C1—C2	49.9 (5)	N3—C8—C9—C10	−6.3 (5)
C6—C1—C2—C3	0.5 (6)	N3—C8—C9—C14	171.1 (3)
N1—C1—C2—C3	176.5 (4)	C14—C9—C10—C11	−1.3 (5)
C1—C2—C3—C4	0.5 (6)	C8—C9—C10—C11	176.1 (3)
C2—C3—C4—C5	−0.2 (7)	C9—C10—C11—C12	0.4 (5)
C3—C4—C5—C6	−1.2 (7)	C10—C11—C12—C13	0.6 (5)
C2—C1—C6—C5	−2.0 (6)	C10—C11—C12—Cl1	−178.6 (3)
N1—C1—C6—C5	−178.1 (4)	C11—C12—C13—C14	−0.5 (6)
C4—C5—C6—C1	2.3 (6)	Cl1—C12—C13—C14	178.7 (3)
C1—N1—C7—N2	−177.2 (3)	C12—C13—C14—C9	−0.5 (6)
C1—N1—C7—S1	4.1 (5)	C10—C9—C14—C13	1.3 (5)
N3—N2—C7—N1	4.1 (4)	C8—C9—C14—C13	−176.1 (3)

### Hydrogen-bond geometry (Å, °)

**Table:**

*D*—H···*A*	*D*—H	H···*A*	*D*···*A*	*D*—H···*A*
N1—H1n···N3	0.85 (2)	2.16 (4)	2.601 (4)	112 (3)
N2—H2n···S1^i^	0.86 (3)	2.57 (3)	3.401 (3)	164 (2)

Symmetry codes: (i) −*x*+1, −*y*, −*z*.
